# Axillary vein thrombosis 30 h after caesarean section: a case report and literature review

**DOI:** 10.1186/s12884-022-05122-y

**Published:** 2022-10-20

**Authors:** Benshuo Cai, Gang Li

**Affiliations:** 1 Department of Obstetrics and Gynecology, Shengjing Hospital of China Medical University, Shenyang, China; 2 Department of Ultrasound, Shengjing Hospital of China Medical University, Shenyang, 110004 China

**Keywords:** Case report, Axillary vein thrombosis, Deep vein thrombosis, Caesarean section, Low-molecular-weight heparin

## Abstract

**Background:**

Axillary vein thrombosis is a very rare disorder. However, a case of axillary vein thrombosis shortly after caesarean section has not been reported previously. We report a case of axillary vein thrombosis 30 h after caesarean section due to an unidentified aetiology.

**Case:**

A 37-year-old multiparous woman developed swelling and pain of the forearm and hand 30 h after undergoing a caesarean section. Doppler ultrasonography revealed a 14.9 mm × 5.3 mm thrombosis in the left axillary vein although a prophylaxis of anticoagulation was administrated. After an adjusted-dose of low-molecular-weight heparin (LMWH) was administered, the patient recovered and was discharged.

**Conclusion:**

Obstetricians should be fully aware of the possibility of upper extremity deep vein thrombosis (DVT) despite its rarity, especially after the surgery. Lying in the lateral decubitus position for long periods postoperatively should be avoided as much as possible.

## Introduction

Upper extremity deep vein thrombosis (DVT) is a rare disorder with an annual incidence of approximately 1–2/100,000, and only 1–4% of all cases of DVT involve the upper extremities [1]. Axillary vein thrombosis is rarer, accounting for 5–25% of upper extremity DVT cases [2]. The aetiology of axillary vein thrombosis is diverse, but physical effort and local trauma have been reported as its main causes. During pregnancy and in the postpartum period, the incidence of lower extremity DVT increases due to a series of physiological and anatomical changes, including hypercoagulability, increased venous stasis, decreased venous outflow, compression of the inferior vena cava and pelvic veins by the enlarging uterus, and decreased mobility, which is five times more than the non-pregnant state [3–8]; however, upper extremity DVT is less likely to occur. A literature review revealed that no case of axillary vein thrombosis during the postpartum period has been previously reported. Here, we report a case of axillary vein thrombosis 30 h after caesarean section.

## Case description

A 37-year-old woman, gravida 2, para 1, with a complaint of “blood pressure increased for 3 months” was admitted to the hospital at 37 weeks’ gestation. Her first delivery had occurred via a caesarean section because of a breech birth. Her current pregnancy had been unremarkable until 24 weeks’ gestation when she developed pregnancy induced hypertension and was treated with oral labetalol hydrochloride. She was also diagnosed with gestational diabetes mellitus at 28 weeks’ gestation; however, her prenatal examinations were irregular, and her blood glucose level was poorly controlled. On admission, a diagnosis of preeclampsia and gestational diabetes at 37 weeks’ gestation was made. After all post-admission evaluations, the patient delivered a 3,270 g healthy male neonate through a caesarean section. Intermittent pneumatic compression was applied to the patient’s lower extremities immediately after delivery, and prophylactic-dose low-molecular-weight heparin (LMWH) (Enoxaparin, 40 mg once daily) [9] was administrated subcutaneously after surgery in view of the various high-risk factors for DVT.

**Fig. 1:**
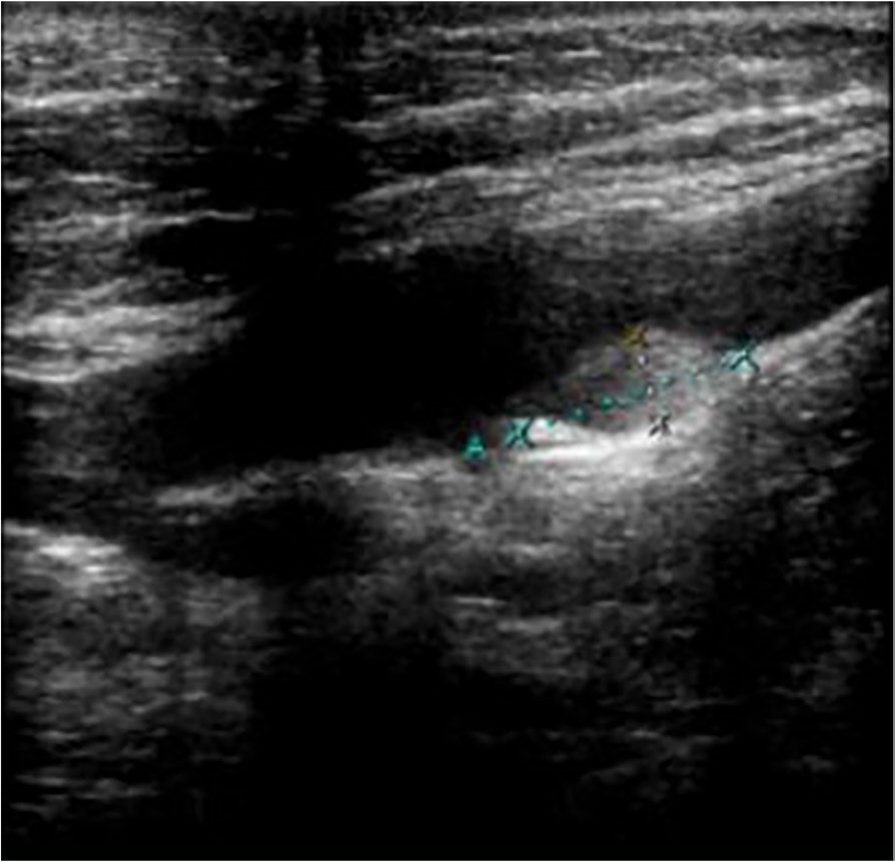
The axillary vein thrombosis on the longitudinal section of the ultrasound

The patient suddenly developed swelling and pain of the forearm and hand 30 h postoperatively, and the elbow joint could not be flexed. The pain increased when the elbow joint was passively flexed. There was no history of trauma, infection, intravenous cannulation, or a history of DVT. Although the patient denied having a familial history of thrombophilia, she never underwent any examination related to thrombophilia. Doppler ultrasound of the left upper extremity arteries and deep veins showed that the left upper extremity axillary vein was dilated, with spontaneous echo reflexes visible in the area, and a weak echo of 14.9 mm × 5.3 mm could be seen next to the local venous valve, where the blood flow was filled with defect (Fig. 1). To rule out an abnormality of the pulmonary arteries and the thoracic outlet, we performed enhanced pulmonary artery computed tomography but no definite abnormality was detected. A thromboelastogram revealed normal findings. Blood coagulation and routine blood tests showed the following results: prothrombin time, 10.8 s; activated partial thromboplastin time, 27 s; D-dimer, 409 µg/L (range, 0–252 µg/L) (the level of D-dimer during the pregnancy fluctuates from 192–243 µg/L); white blood cells count, 12.8 × 10^9^/L; haemoglobin, 124 g/L; and haematocrit, 37.97%. To rule out thrombophilia, we planned to perform thrombophilia-related examinations, including factor V Leiden, prothrombin 20210A mutation, protein C, protein S, and antiphospholipid antibody, but the patient declined the investigations due to financial constraints. Based on clinical manifestations and imaging findings, we made a diagnosis of left axillary vein thrombosis. We immediately started administering an adjusted-dose of LMWH (Enoxaparin, 1 mg/kg 12 hourly) subcutaneously [9] and kept her left arm completely immobilised to prevent embolisation into the pulmonary artery or other fatal locations. On the fifth day of treatment, the swelling resolved, and the pain disappeared. Doppler ultrasonography of the deep veins of the left upper extremity showed that the thrombosis had disappeared. Her D-dimer level decreased to 344 µg/L. The patient was discharged and asked to continue anticoagulant therapy for 6 weeks. A Doppler ultrasound was performed on follow-up every month for 3 months; however, no abnormality was detected.

## Discussion

To the best of our knowledge, this is the first report of a case of axillary vein thrombosis shortly after caesarean section in the literature. From a review of relevant literature, we found only six reports of axillary vein thrombosis related to pregnancy. One patient had an inherited deficiency of antithrombin III [10]; one case was related to the presence of a circulating lupus anticoagulant [11]; one patient was heterozygous for both the prothrombin 3′ UTR mutation and factor V Leiden mutation and presented with axillary vein thrombosis following in vitro fertilisation [12]; the fourth case was related to strenuous exercise of the upper extremity [13]; and no aetiology could be identified in the other two cases [14, 15]. All occurrences of axillary vein thrombosis in these reports were before delivery.

Upper extremity DVT is relatively rare, especially during pregnancy and the postpartum period. According to the pathogenesis, upper extremity DVT is classified as primary or secondary. The primary type was originally described by James Paget in 1875 and Leopold van Schrotter in 1884, and is mainly related to anatomical abnormalities of the thoracic outlet and strenuous upper extremity exercise; it accounts for approximately 20% of all cases of upper extremity DVT [16]. The secondary type is mainly associated with central venous catheterisation, phlebitis, intravenous infusion, trauma, infection, use of pacemakers, malignancy, congestive heart failure, and a hypercoagulable state (thrombophilia) [17, 18]; it accounts for 80% of all cases of upper extremity DVT. In the present case, abnormality of the thoracic outlet and strenuous upper extremity exercise were both excluded; therefore, it was not a primary type. Instead, the patient had various high-risk factors for DVT, such as hypercoagulable state due to pregnancy, advanced age, multiparity, obesity (body mass index, 31.25 kg/m^2^), diabetes mellitus, preeclampsia, and undergoing a caesarean section [3, 19–29]. As the patient did not exhibit any symptoms before surgery, we speculate that the surgery may have induced thrombosis. As intermittent pneumatic compression was applied to the patient’s lower extremities immediately after surgery, thrombosis did not occur in the lower extremities. However, the patient was used to lying on her left side during the third trimester of pregnancy, and the left lateral decubitus position was maintained postoperatively. Hariri reported a case of upper extremity DVT associated with lying in the lateral decubitus position [30]. We speculate that the combination of high-risk factors and lying in the left lateral decubitus position postoperatively led to the occurrence of thrombosis in this patient. Nevertheless, other possible contributing factors, such as thrombophilia, unnoticed thrombophlebitis in one of the left peripheral veins where the intravenous access had ever been placed, or the neglected prolonged hyperextension of the upper extremity during the caesarean section, could not be completely excluded. Paradoxically, the patient had been administered a prophylactic dose of LMWH after surgery.

Although upper extremity DVT is a very rare condition, it should be given enough consideration. For pregnant women with high-risk factors for thrombosis, obstetricians should consider the possibility of upper extremity DVT despite its rarity, especially after surgery. Furthermore, prolonged lying in the lateral decubitus position postoperatively should be avoided as much as possible. In addition, we acknowledge that there is a limitation to this case that the thrombophilia-related examinations were not performed so that the aetiology of the upper extremity DVT in this case was not identified more clearly, further thrombophilia-related examinations and a consecutive follow-up should be performed in the future.

## Data Availability

The original contributions presented in the study are included in the article, further inquiries can be directed to the corresponding author.

## Abbreviation

DVT: Deep vein thrombosis
